# Virtual Reality vs. Tablet Video as an Experiential Education Platform for Pediatric Patients Undergoing Chest Radiography: A Randomized Clinical Trial

**DOI:** 10.3390/jcm10112486

**Published:** 2021-06-04

**Authors:** Jung-Hee Ryu, Jin-Woo Park, Sang Il Choi, Ji Young Kim, Hyunju Lee, Hee-Jeong Yoo, Sung-Hee Han

**Affiliations:** 1 Medical Virtual Reality Research Group, Department of Anesthesiology and Pain Medicine, Seoul National University College of Medicine, Gwanak-gu, Seoul 03080, Korea; jinaryu74@gmail.com (J.-H.R.); jinul8282@gmail.com (J.-W.P.); 2 Department of Anesthesiology and Pain Medicine, Seoul National University Bundang Hospital, Bundang-gu, Seongnam 13620, Korea; 3 Medical Virtual Reality Laboratory, Seoul National University Bundang Hospital, Bundang-gu, Seongnam 13620, Korea; drsic@snubh.org (S.I.C.); jiyoungkim@snubh.org (J.Y.K.); mdopd@hanmail.net (H.L.); hjyoo@snubh.org (H.-J.Y.); 4 Department of Radiology, Seoul National University College of Medicine, Seoul National University Bundang Hospital, Bundang-gu, Seongnam 13620, Korea; 5 Department of Pediatrics, Seoul National University College of Medicine, Seoul National University Bundang Hospital, Bundang-gu, Seongnam 13620, Korea; 6 Medical Virtual Reality Research Group, Department of Psychiatry, Seoul National University College of Medicine, Seoul National University Bundang Hospital, Bundang-gu, Seongnam 13620, Korea

**Keywords:** anxiety, chest radiography, distress, education, virtual reality

## Abstract

Virtual reality (VR), which offers an immersive experience, has been implemented into the education of pediatric patients to reduce peri-procedural anxiety. This randomized clinical trial evaluated the effect of VR, compared with standard video, on reducing anxiety and distress in pediatric patients undergoing chest radiography. A total of 120 children aged 4 to 8 years with scheduled chest radiography appointments were randomized into either the tablet or the VR group. Children in the tablet group experienced chest radiography indirectly with a 3 min tablet video, whereas those in the VR group received the same content via a VR experience. The distress of children was measured using the Observational Scale of Behavioral Distress (OSBD) scale. Parental presence and procedural outcomes were also recorded. The number of less distressed children (OSBD score < 5) was significantly higher in the VR group than in the tablet group (49 [81.7%]) vs. 32 [53.3%]) (*p* = 0.001). The OSBD scores, the need for parental presence, the procedure time, and the number of repeated procedures were all lower in the VR group. The immersive VR experience appears to decrease the degree of anxiety in children and increase the efficiency of the procedures compared with the tablet video with the same content.

## 1. Introduction

Virtual reality (VR) provides a realistic and immersive experience on a digital platform by placing the user inside a three-dimensional world. Video, on the other hand, utilizes a screen, lacking the three-dimensional and immersive aspects of VR. The immersive and interactive characteristics of VR are proven to be useful in a variety of domains of medical education. In addition to healthcare education, immersive VR is used in patient treatment, including cognitive rehabilitation and exposure therapy for anxiety disorders [1,2].

Pediatric patients usually suffer from anxiety and distress ahead of a procedure or surgery; the primary challenge of hospital staff is to reduce such anxiety and distress. Besides various interventions, such as premedication, parental presence and behavioral intervention [3,4], exposure therapy using video or VR is shown to be effective in controlling anxiety and distress among children before a procedure or surgery [5,6,7,8]. Exposure using video or VR allows the pediatric patients to confront the fearful or unknown situation by providing visual expectations ahead of the procedure, allowing the children to become familiar with the room and the overall process [1].

There have been many studies on how far VR reduces distress and anxiety in pediatric patients during perioperative or periprocedural period [5,6,8,9]. VR has been shown to be superior in reducing anxiety in children compared to the conventional method, which only provides verbal information on what to expect. However, to date, it remains unknown whether the use of VR itself may decrease children’s anxiety in hospitals. It is hypothesized that VR itself offers a novel solution for decreasing peri-procedural anxiety in children who are susceptible to stress. Therefore, this study evaluates the effect of VR, compared with standard video, on reducing anxiety and distress in pediatric patients undergoing chest radiography.

## 2. Materials and Methods

### 2.1. Study

The institutional review board of Seoul National University Bundang Hospital approved the protocol of this prospective randomized controlled study (No: B-1901/514-304), which was registered at University Hospital Medical Information Network (UMIN) Clinical Trials Registry (registration number: UMIN000035485). All parents or guardians agreed and signed the written informed consent. Additionally, pediatric patients aged 7 years or older received detailed instructions about this protocol and signed additional agreements with their parents or guardians. This study was performed at the Seoul National University Bundang Hospital between 10 January 2019 and 22 March 2019. Statistical analysis was carried out from 2 August 2019 to 5 September 2019.

### 2.2. Patients

Children aged 4 to 8 years who were scheduled for chest radiography were enrolled in this study. The exclusion criteria were children with: (1) visual or hearing impairment; (2) cognitive deficits or cognitive and intellectual developmental disabilities; (3) a history of prematurity or congenital disease; (4) a history of epilepsy or seizures, or taking psychoactive medications; and (5) prior experience of chest radiography during the past year.

### 2.3. Randomization

Randomization was performed by an independent researcher who was only in charge of patient allocation 10 min before chest radiography. Children were randomized to either the tablet or VR group via a computer-generated randomization code (Random Allocation Software, version 1.0; Isfahan University of Medical Sciences). An opaque envelope containing sequential numbers was delivered to another researcher, who performed the intervention in a separated area 5 min before entering the radiography room. During chest radiography, the blinded assessor observed the child and made an evaluation based on the Observational Scale of Behavioral Distress (OSBD) scale [10] in the radiography room. The assessor and radiology technologist were blinded to the allocation, whereas children and their parents were not blinded.

### 2.4. Intervention

**Figure 1:**
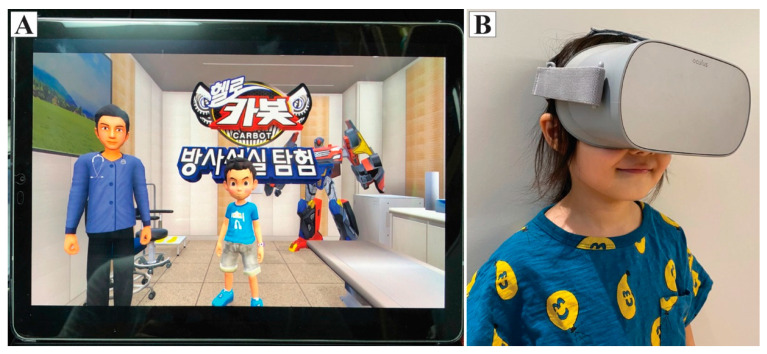
Experiential education platforms. (**A**) Tablet video. (**B**) Virtual reality experience with a head-mounted display.

**Figure 2:**
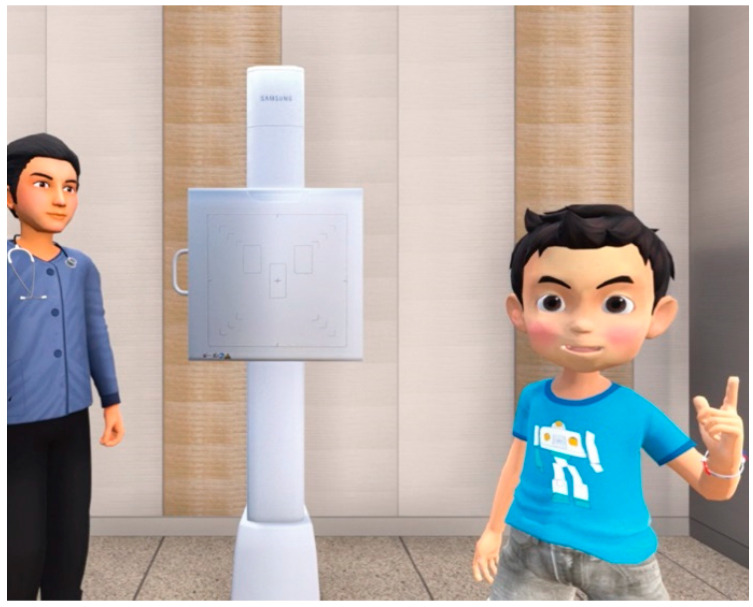
Chatan explaining how to cooperate appropriately for chest radiography.

Children in the tablet group experienced the process of chest radiography indirectly with a 3 min video using a tablet PC (Figure 1A). Children in the VR group, on the other hand, experienced the same content and information as the control group, but using a head-mounted VR display (Figure 1B), offering a 360°, 3-dimensional virtual environment that introduced and explained the process of chest radiography, which was described by Han et al. [5]. In this VR content, the participant, along with Chatan and Ace, who are famous animation characters in the animated film *Hello Carbot* (Choi- Rock Contents Factory, Seoul, Republic of Korea), experienced the chest radiography together in the radiography room (Figure 2). This VR content was converted to the tablet video, which was provided to those in the tablet group.

### 2.5. Outcome Measurement

For consistency across outcomes, one blinded observer evaluated the degree of stress and anxiety among all children during the radiography process. The amended version of the OSBD scale for radiographic procedures was used to measure the degree of distress among the children in this study [5]. The OSBD scale (total scale of 30) consists of 11 operationally defined behaviors indicative of distress, including crying, clinging, fear, restraint and screaming [10]. In the current study, the OSBD scores of children were classified into 2 categories (more distressed and less distressed) with a cut-off score of 5, based on a previous study that used the amended OSBD score. ‘Less distressed’ was defined as an OSBD score of less than 5 [5,11].

The blinded radiology technologist asked the children if they wanted to get the chest radiography with their parent or guardian; based on the response, the parental presence was counted. Parental satisfaction score was recorded based on an 11-point numerical rating scale (NRS; 0, dissatisfied; 10, satisfied) right after the chest radiographic process.

The duration of the radiographic procedure (from entrance to the radiography room to the production of a chest radiographic image) and the number of repeated procedures (re-take) were measured by a blinded single evaluator. The radiography technologist subjectively scored the degree of easiness for the procedure using NRS (0, very difficult; 10, very easy) immediately after the chest radiography.

### 2.6. Statistical Analysis

G*Power software, version 3.1.2 (Heinrich Heine University) was used for power analysis. Sample size calculation was based on the result of the pilot study with 20 children in the tablet group. They showed the OSBD of 4 (3.7) during chest radiography. A decrease of 50% in the OSBD mean score by VR experience was considered to be clinically significant, and a sample size of 60 children per group was calculated (power of 0.8, significance level of 0.05) with a 10% dropout rate.

In the current study, all statistical analyses were conducted using SPSS, version 21.0 (SPSS Inc., Chicago, IL, USA) and intention-to-treat (ITT) was used for data analysis. The Mann–Whitney *U* test was used for continuous variables, which are presented as the median (IQR). The χ^2^ test or Fisher’s exact test were then used to analyze categorical variables, which are shown as a number (percentage). All of the reported *p* values were 2-sided, and *p* < 0.05 was considered to indicate statistical significance.

## 3. Results

**Figure 3:**
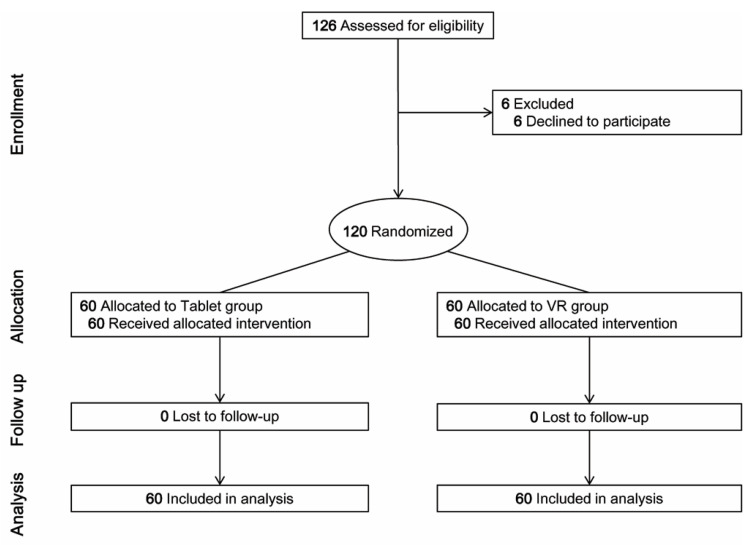
CONSORT Flow Diagram. VR, virtual reality.

One hundred and twenty-six children were screened for this study, and six children were excluded from the randomization due to parental refusal. A final total of 120 children (tablet group: 60, VR group: 60) participated in this study; no one was withdrawn during the study period (Figure 3). Patients’ characteristics were similar between the two groups (Table 1). There were two reasons for chest radiography: respiratory and/or cardiovascular symptoms (21 in the tablet group; 16 in the VR group), or preoperative workup (48 in the tablet group; 44 in the VR group). All of the analysis performed in this study can be seen in Table 2 and Table 3.

The number of less distressed children (OSBD score, <5) was significantly higher in the VR group than in the tablet group (49 (81.7%)) vs. 32 (53.3%)) (*p* = 0.001). In addition, the OSBD score was lower in the VR group than in the tablet group (median (IQR) OSBD score, 1.0 (0.8–3.0) vs. 4.0 (2.0–7.0); *p* < 0.001) (Table 2).

The need for parental presence was lower in the VR group than in the tablet group (5 [8.3%] vs. 19 [31.7%]; *p* = 0.001, Table 2). However, parental satisfaction scores were comparable between the two groups (*p* = 0.599, Table 2).

The total procedure time for the chest radiography was significantly less in the VR group than in the tablet group (48.0 (43.0–54.3) s vs. 65.0 (53.8–74.5) s; *p* < 0.001, Table 3). The number of repeated chest radiographic procedures was two (3.3%) in the VR group and six (10%) in the tablet group, although this difference was not statistically significant (Table 3). The degree of easiness during the chest radiography was scored by the radiologic technologists; the degree of easiness was higher in the VR group than in the tablet group (10.0 (10.0–10.0) vs. 8.0 (7.0–9.0); *p* < 0.001, Table 3).

## 4. Discussion

To the best of our knowledge, this is the first study to evaluate the effect of VR on peri-procedural distress in children and the efficiency of the procedure compared with the table video using the same content. The results of the current study suggest that pre-procedural education using VR may significantly reduce anxiety and distress in children and may also reduce the need for parental presence during the chest radiography compared with the tablet video. In addition, the VR experience improved the efficiency of the procedure by reducing the overall duration of the procedure and the need of repeated takes.

Anxiety and distress in children before chest radiography was measured using the OSBD scores; the OSBD scores in the VR group were significantly lower than those in the tablet group. As aforementioned, the OSBD scores were classified into two groups (more distressed and less distressed), and there were more children who were less distressed in the VR group than in the tablet group. Moreover, parental presence was less required in the VR group than in the tablet group. Previous studies with respect to the effect of VR on preoperative anxiety also revealed that children who received education using VR prior to undergoing a procedure or surgery had a reduced degree of anxiety and distress [5,6,8,9]. It is worth noting that previous studies evaluating the effect of VR were limited to only comparing VR with the conventional verbal education. The current study, however, compared the effect of VR with that of video using tablet PCs that displayed the same content [5,6,8,9].

Distress and anxiety in children have a great influence on the efficiency of many hospital procedures. In the current study, VR exposure and education in children prior to undergoing the procedure reduced the overall duration of the radiographic procedure by about 23%, while simultaneously increasing the easiness to perform the procedure by the radiology technologist. In a previous investigation with the same procedure, the control group with conventional verbal education needed about 75 s [5], whereas the tablet group with the same content as the VR used for this study required around 66.5 s. The VR group in both studies required around 55 s [5]. The occurrence of a repeated procedure was less frequent in the VR group than in the tablet group, although statistical difference was not reached. These results suggest that VR itself may improve the efficiency of the procedure.

The pediatric patients in the VR group experienced the immersive VR system with a head-mounted display, isolated from the real world. In a 360° and 3-dimensional world designed to exactly simulate a real radiology room, they interacted with the virtual environment; these elements seemed to enhance immersion and improve educational quality [12]. Although it has gained more attention across various fields [13,14,15], VR is still a less common technology. A new and exciting technology might have positively influenced the chest radiography experience [16]. While there is no way to avoid this confounding effect for now, the potential effect of novelty should be taken into account.

In this study, chest radiography, which is a relatively simple and short procedure, was chosen because it is one of the most common radiographic procedures. However, the level of anxiety and distress should not be ignored in children even in this simple and short procedure. Additionally, the management of anxiety and distress in children is required especially in procedures that may be longer or more invasive, i.e., needing sedation [17,18]. More studies regarding the efficacy of VR education with longer or more invasive pediatric procedures, such as pediatric vascular interventional radiology, computed tomography and magnetic resonance imaging, are needed to explore the application of this VR technology to a broader field of medicine.

In conclusion, this study investigated the effect of VR on distress and anxiety in children undergoing chest radiography by comparing the two modes of education delivery: the VR experience and the non-VR standard tablet video experience. The immersive and vivid VR experience ahead of chest radiography decreased the anxiety in children and increased the efficiency of the procedure. These results suggest that VR may be a feasible exposure therapy to reduce anxiety and distress, with the potential of widespread applications in the field of medicine.

## Figures and Tables

**Table 1:** Patients’ characteristics.

	Tablet Group (*n* = 60)	VR Group (*n* = 60)
Age (years)	6.0 (4.8–7.0)	6.0 (5.0–7.3)
Male	25 (41.7)	32 (53.3)
Weight (kg)	20.6 (18.2–26.4)	22.3 (18.9–28.2)
Height (cm)	113.8 (106.7–124.4)	118.3 (109.8–127.8)
Reason for chest radiography		
respiratory or cardiovascular symptom	12 (20.0)	16 (26.7)
preoperative work up	48 (80.0)	44 (73.3)

Values are presented as median (IQR) or number (%). VR, virtual reality.

**Table 2:** Distress of pediatric patients, the need of parental presence and parental satisfaction during chest radiography.

	Tablet Group (*n* = 60)	VR Group (*n* = 60)	Risk Ratio (95% CI)	*p*-Value
OSBD group				
More Distressed (≥5)	28 (46.7)	11 (18.3)	3.9 (1.7–8.9)	0.001
Less distressed (<5)	32 (53.3)	49 (81.7)		
OSBD score	4.0 (2.0–7.0)	1.0 (0.8–3.0)		<0.001
Parental presence	19 (31.7)	5 (8.3)	5.1 (1.8–14.8)	0.001
Parental satisfaction score	10.0 (9.0–10.0)	10.0 (9.0–10.0)		0.599

Values are presented as median (IQR) or number (%). VR, virtual reality; OSBD, Observational Scale of Behavioral Distress; CI, confidential interval.

**Table 3:** Total procedure time, the number of repeated procedure and easiness score during chest radiography.

	Tablet Group (*n* = 60)	VR Group (*n* = 60)	Risk Ratio (95% CI)	*p*-Value
Time for radiography procedure (s)	65.0 (53.8–74.5)	48.0 (43.0–54.3)		<0.001
Repeated procedures	6 (10.0)	2 (3.3)	3.2 (0.6–16.7)	0.272
Easiness score for the procedure	8.0 (7.0–9.0)	10.0 (10.0–10.0)		<0.001

Values are presented as median (IQR) or number (%). VR, virtual reality; CI, confidential interval.

## Data Availability

The datasets generated and analyzed during the current study are available from the corresponding author on reasonable request.
